# Supplementary material for: Magnetic resonance measurements of cellular and sub-cellular membrane structures in live and fixed neural tissue
Source: eLife. 2019 Dec 12;8:e51101. doi: 10.7554/eLife.51101 (PMC6977971; doi:10.7554/eLife.51101)
Supplement: Transparent reporting form [file elife-51101-transrepform.docx]

***eLife’s* transparent reporting form**

We encourage authors to provide detailed information *within their submission* to facilitate the interpretation and replication of experiments. Authors can upload supporting documentation to indicate the use of appropriate reporting guidelines for health-related research (see [EQUATOR Network](http://www.equator-network.org/%20)), life science research (see the [BioSharing Information Resource](https://biosharing.org/)), or the [ARRIVE guidelines](http://www.plosbiology.org/article/info:doi/10.1371/journal.pbio.1000412) for reporting work involving animal research. Where applicable, authors should refer to any relevant reporting standards documents in this form.

If you have any questions, please consult our Journal Policies and/or contact us: [editorial@elifesciences.org](mailto:editorial@elifesciences.org).

**Sample-size estimation**

- You should state whether an appropriate sample size was computed when the study was being designed
- You should state the statistical method of sample size computation and any required assumptions
- If no explicit power analysis was used, you should describe how you decided what sample (replicate) size (number) to use

Please outline where this information can be found within the submission (e.g., sections or figure legends), or explain why this information doesn’t apply to your submission:

This exploratory pilot study, which involved the development of novel methods of measuring tissue microstructure, did not seek to test a hypothesis. Therefore, sample size estimation is not appropriate. However, the data generated here can be used to estimate an effect size such that a power analysis can be performed for future studies.

**Replicates**

- You should report how often each experiment was performed
- You should include a definition of biological versus technical replication
- The data obtained should be provided and sufficient information should be provided to indicate the number of independent biological and/or technical replicates
- If you encountered any outliers, you should describe how these were handled
- Criteria for exclusion/inclusion of data should be clearly stated
- High-throughput sequence data should be uploaded before submission, with a private link for reviewers provided (these are available from both GEO and ArrayExpress)

Please outline where this information can be found within the submission (e.g., sections or figure legends), or explain why this information doesn’t apply to your submission:

Nuclear Magnetic Resonance measures signal from the ensemble of proton spins within the sample and is therefore averaged to begin with. In general, deviations on the same sample should simply be due to the signal to noise ratio. Repeated measurements were performed on the same sample to provide standard deviations for estimated parameters with respect to system noise. Measurements were performed on multiple samples to measure the deviation in measurements from sample-to-sample due to biological variation. All instances of error bars, standard deviations, and confidence intervals are explained as coming from repeated measurements on the same sample (e.g., “Error bars are the standard deviations from three repeat measurements.”), or from multiple samples (n=x). We explore the sources of measurement variability with regards to repeat measurement variability and sample-to-sample variability in **Figure 2–Figure supplement 1, Figure 4–Figure supplement 1, and Figure 6–Figure supplement 1.**

The number of repeated measurements is provided in the figure captions.

Information on sample size can be found in **Methods** subsection **Statistical analysis and reproducibility.**

**Statistical reporting**

- Statistical analysis methods should be described and justified
- Raw data should be presented in figures whenever informative to do so (typically when N per group is less than 10)
- For each experiment, you should identify the statistical tests used, exact values of N, definitions of center, methods of multiple test correction, and dispersion and precision measures (e.g., mean, median, SD, SEM, confidence intervals; and, for the major substantive results, a measure of effect size (e.g., Pearson's r, Cohen's d)
- Report exact p-values wherever possible alongside the summary statistics and 95% confidence intervals. These should be reported for all key questions and not only when the p-value is less than 0.05.

Please outline where this information can be found within the submission (e.g., sections or figure legends), or explain why this information doesn’t apply to your submission:

We present raw data from individual measurements (Figures 1, 3, and 7), means and standard deviations from repeated measurements on the same specimen (Figures 2, 4, and 5) as well as means and standard deviations from measurements on multiple samples (Figs. 6 and 8). Anderson-Darling tests of Gaussian variability were performed and p values are reported in Appendix 2.

(For large datasets, or papers with a very large number of statistical tests, you may upload a single table file with tests, Ns, etc., with reference to sections in the manuscript.)

**Group allocation**

- Indicate how samples were allocated into experimental groups (in the case of clinical studies, please specify allocation to treatment method); if randomization was used, please also state if restricted randomization was applied
- Indicate if masking was used during group allocation, data collection and/or data analysis

Please outline where this information can be found within the submission (e.g., sections or figure legends), or explain why this information doesn’t apply to your submission:

Data was grouped based on the treatment of the spinal cord (e.g., live, fixed, and fixed delipidated).

**Additional data files (“source data”)**

- We encourage you to upload relevant additional data files, such as numerical data that are represented as a graph in a figure, or as a summary table
- Where provided, these should be in the most useful format, and they can be uploaded as “Source data” files linked to a main figure or table
- Include model definition files including the full list of parameters used
- Include code used for data analysis (e.g., R, MatLab)
- Avoid stating that data files are “available upon request”

Please indicate the figures or tables for which source data files have been provided:

Source data for all Figures 1-9 in the manuscript have been provided as supporting files. Novel NMR pulse sequences and analysis routines are provided as supporting files.
